# Pretreatment Gastric Lavage Reduces Postoperative Bleeding after Endoscopic Submucosal Dissection for Gastric Neoplasms

**DOI:** 10.1371/journal.pone.0149235

**Published:** 2016-02-12

**Authors:** Hiroyuki Nakanishi, Masayuki Kurosaki, Yuka Takahashi, Jun Itakura, Ken Ueda, Shoko Suzuki, Yutaka Yasui, Nobuharu Tamaki, Natsuko Nakakuki, Hitomi Takada, Masako Ueda, Tsuguru Hayashi, Konomi Kuwabara, Kenta Takaura, Mayu Higuchi, Yasuyuki Komiyama, Tsubasa Yoshida, Namiki Izumi

**Affiliations:** Department of Gastroenterology and Hepatology, Musashino Red Cross Hospital, Tokyo, Japan; Chiba University, Graduate School of Medicine, JAPAN

## Abstract

**Aim:**

For patients receiving endoscopic submucosal dissection (ESD), there is urgent need pertaining to the prevention of postoperative bleeding. We conducted a retrospective propensity score-matched study that evaluated whether pre-ESD gastric lavage prevents postoperative bleeding after ESD for gastric neoplasms.

**Methods:**

From September 2002 to October 2015, the 760 consecutive patients receiving ESD for gastric neoplasm were enrolled and data regarding them were retrospectively analyzed. All patients received conventional preventive treatment against delayed bleeding after ESD, including the administration of proton pump inhibitor and preventive coagulation of visible vessels, at the end of the ESD procedure.

**Results:**

Pre-ESD risk factors for postoperative bleeding included tumor size and no gastric lavage. Using multivariate analysis tumor size >2.0 cm (HR 2.90, 95% CI 1.65–5.10, p = 0.0002) and no gastric lavage (HR 3.20, 95% CI 1.13–9.11, p = 0.029) were found to be independent risk factors. Next, we evaluated the effect of gastric lavage on the prevention of post-ESD bleeding using a propensity score-matching method. A total of 284 subjects (142 per group) were selected. Adjusted odds ratio of gastric lavage for post-ESD bleeding was 0.25 (95% CI 0.071–0.886, p = 0.032).

**Conclusion:**

Pretreatment gastric lavage reduced postoperative bleeding in patients receiving ESD for gastric neoplasm.

## Introduction

Endoscopic submucosal dissection (ESD) is widely performed as a standard endoscopic treatment for gastric neoplasms having negligible probability of lymph node metastasis because of its high rate of curability [1–8]. But ESD creates a large artificial ulcer, thereby ESD is associated with a higher risk of complications than endoscopic mucosal resection [2, 4, 9–12]. The reported incidence of postoperative bleeding is as high as 0–15.6% [13], and endoscopic hemostasis during emergency endoscopy is effective for stopping the bleeding. The bleeding after ESD causes hematemesis or melena which may result in severe anemia and circulatory failure. Therefore, there is urgent need for prevention of postoperative bleeding. Otherwise there were some studies reported that pyrexia (defined as a body temperature above 37.5 °C) occurred at an incidence of  6  - 24.8 % after gastric ESD [14–16]. Recently there was a report that showed gastric irrigation with saline solution suppress local infection during the ESD procedure[17] and prevent pyrexia after ESD. However, the preventive effect of pretreatment gastric lavage on bleeding after ESD was not studied. We conducted retrospective propensity score matched study that evaluated whether pre-ESD gastric lavage prevent bleeding after ESD.

## Patients and Methods

The consecutive 760 ESD procedures for gastric neoplasm patients from September 2002 to October 2015 were enrolled and retrospectively analyzed. All patients began receiving a proton pump inhibitor at least 1week prior to ESD. Preventive coagulation of visible vessels in the artificial ulcer base was performed in all cases at the end of ESD procedure. Postoperative bleeding was defined as hematemesis or melena or Hb > 2 g/dL decrease 6 hours or more after the end of ESD which required emergency endoscopic hemostasis and evident bleeding sites on resected lesions was confirmed. Subjects were divided into a gastric lavage group; in which gastric lavage was performed before ESD, and no gastric lavage group; in which gastoric lavage was not performed. From September 2002 to March 2015, we only washed the lesion with 40 to 60 mL of tap water. From April 2014 to October 2015, pretreatment gastric lavage was performed by 1L of tap water injected though the scope in consecutive 148 patients receiving ESD. We performed gastric lavage just before indigo carmine spraying and marking during ESD proceidure. And we endoscopically aspirated and drained the washing water. We first analyzed the pretreatment risk factors for postoperative bleeding after ESD performance. After that we investigated whether gastric lavage could reduce the postoperative bleeding by matching factors other than gastric lavage with propensity score matching method. The protocol was undertaken in accordance with the World Medical Association’s Declaration of Helsinki and was approved by the institutional review board of Musashino Red Cross Hospital, Tokyo, Japan (No. 766). Written informed consent was given by participants for their clinical records to be used in this study.

### Statistical Analysis

Values for continuous variables were presented as means ± standard deviation. The paired or non-paired Student t test was used to assess the significance of the differences in the comparison of continuous data, whereas categorical variables were assessed by the Fisher exact test. P values below .05 in each analysis were considered to be statistically significant. To reduce potential confounding effects due to patient background variability in the direct comparison between gastric lavage group and no gastric lavage group, the propensity score matching method was used. We analyzed with matching factors other than gastric lavage which related to the post-ESD bleeding. These analyses were performed by using SPSS version 17.0 software (SPSS Inc, Chicago, IL) or R software packages, version 2.1.1 (R Development Core Team, Vienna, Austria).

## Results

The completion rate, en bloc resection rate and curative resection rate of ESD was 98.9%, 96.6% and 92.8% respectively. The frequency of complication except for bleeding were microperforation (1.8%) and aspiration pneumonia (2.9%). There was no patient who needed emergent surgery. The total post-ESD bleeding rate in 760 patients was 7.1%. The 54 patients had postoperative bleeding, and 706 patients did not have post operative bleeding. In monovariate analysis the pretreatment risk factors for postoperative bleeding after ESD were tumor size (p <0.001) and without gastric lavage (p = 0.02) (Table 1). And continuous antithrombotic agents was one of risk factors with borderline significance (p = 0.07). And procedure time is significant factor as during or post-ESD factors. In multivariate analysis tumor size > 2.0cm (HR 2.90, 95%C.I. 1.65–5.10, p = 0.0002) and without gastric lavage (HR 3.20, 95%C.I. 1.13–9.11, p = 0.0291) were independent factors associated with post-ESD bleeding (Table 2).

**Table 1:** The comparison between post-ESD bleeding and no bleeding patients.

		bleeding (+)	bleeding (-)	p value
		n = 54	n = 706	
Age		73.59 ± 7.08	72.94 ± 8.67	n.s
Gender; male (%)	n, (%)	38 (70.4)	513 (72.7)	n.s
Performance status ≧1	n, (%)	9 (16.7)	109 (15.4)	n.s
Hemodialysis	n, (%)	3 (5.6)	18 (2.5)	n.s
Cerebral vascular injury	n, (%)	10 (18.5)	115 (16.3)	n.s
Gastric lavage	n, (%)	4 (7.4)	144 (20.4)	0.02
Located upper third portion	n, (%)	8 (14.8)	110 (15.6)	n.s
Tumor size	(cm)	2.43 ±1.46	1.78 ±1.09	<0.001
Submucosal invasion	n, (%)	7 (13.0)	77 (10.9)	n.s
Ulcer or ulcer scar	n, (%)	6 (11.1)	62 (8.8)	n.s
Pathology				n.s
Tubular or papillary adenocarcinoma	n, (%)	39 (72.2)	485 (68.7)	
Poorly differentiated adenocarcinoma or signet-ring cell carcinoma	n, (%)	1 (1.9)	17 (2.4)	
adenoma	n, (%)	14 (25.9)	196 (27.8)	
others	n, (%)	0 (0.0)	8 (1.1)	
Elevated type	n, (%)	34 (63.0)	502 (71.1)	n.s
Procedure time	(min)	143.70 ±91.74	101.15 ±66.83	<0.001
En bloc resection	n, (%)	51 (94.4)	683 (96.7)	n.s
Curative resection	n, (%)	50 (92.6)	655 (92.8)	n.s
Continuous antithrombotic agents	n, (%)	4 (7.4)	19 (2.7)	0.07

**Table 2:** The pretreatment factors associated with post-ESD bleeding.

n = 760	HR	95% C.I	p value
No gastric lavage	3.20	1.13–9.11	0.0291
Tumor size>2.0 cm	2.90	1.65–5.1	0.0002
Continuous antithrombotic agents	3.07	0.948–9.97	0.0614

**Fig 1:**
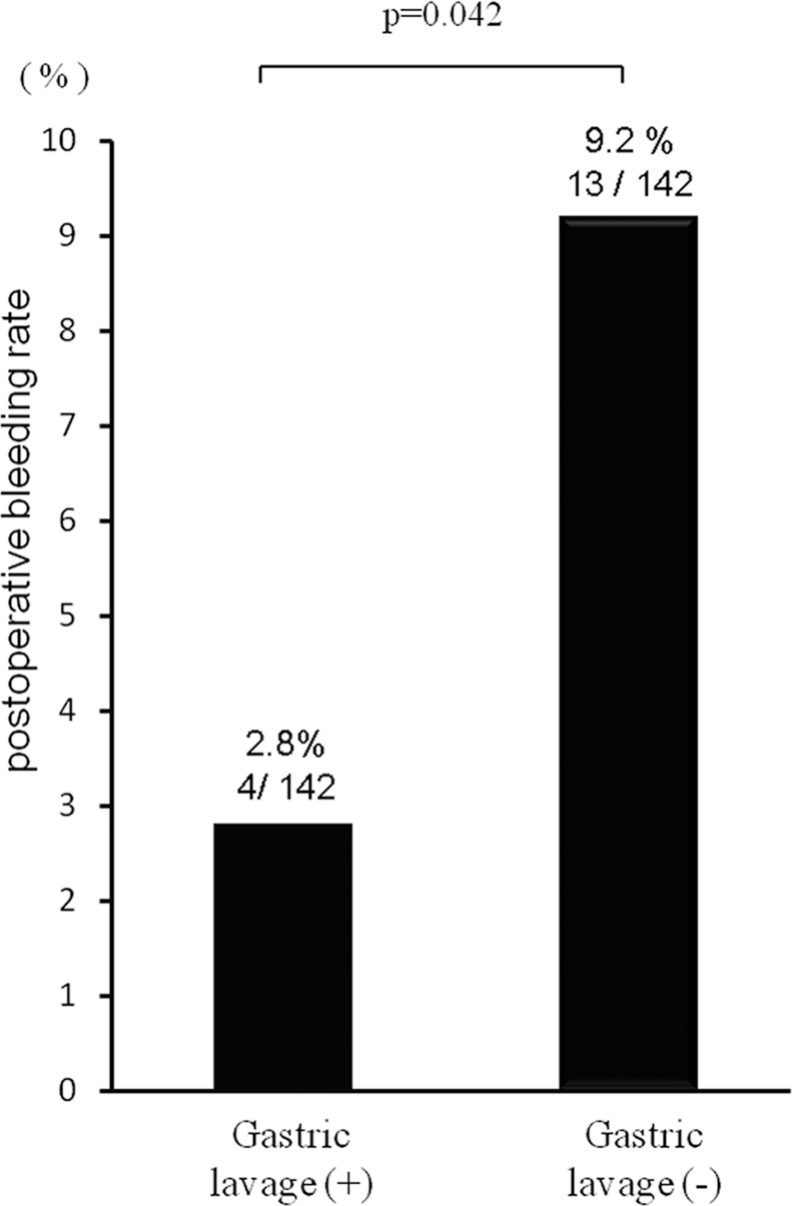
The comparison of postoperative bleeding between gastric lavage group and no gastric lavage group.

So we evaluated whether gastric lavage itself reduces postoperative bleeding on an artificial gastric ulcer in ESD by tumor size matched with propensity score matching method. For propensity score matched analysis, 284 patients (142 in each group) were selected and analyzed (Table 3). Between gastric lavage group (n = 142) and no gastric lavage group (n = 142), the rate of postoperative bleeding after ESD were 2.8% and 9.2% respectively (p = 0.042). And gastric lavage significantly reduced the risk of post-ESD bleeding (adjusted odds ratio 0.25, 95%C.I. 0.071–0.886, p = 0.032) (Fig 1, Table 4).

**Table 3:** Comparison of patients demographics between gastric lavage group and no gastric lavage group.

	Gastric lavage	No gastric lavage	p value
	n = 142	n = 142	
Age	75.1 ± 7.8	72.7 ± 9.4	0.023
Gender; male n, (%)	103 (72.5)	108 (76.1)	0.587
Hemodyalysis n, (%)	6 (4.2)	2 (1.4)	0.282
Cerebral vascular injury n, (%)	20 (14.1)	24 (16.9)	0.623
Located upper third portion n, (%)	16 (11.3)	21 (14.8)	0.481
Submucosal invasion n, (%)	20 (14.1)	14 (9.9)	0.361
Ulcer or ulcer scar (%)	15 (10.6)	17 (12.0)	0.851
Pathology n, (%)			0.396
Tubular or papillary adenocarcinoma	110 (77.5)	104 (73.2)	
Poorly differentiated adenocarcinoma or signet-ring cell carcinoma	0 (0.0)	3 (2.1)	
adenoma	31 (21.8)	33 (23.2)	
others	1 (0.7)	2 (1.4)	
Elevated type n, (%)	95 (66.9)	104 (73.2)	0.3
Tumor size (cm)	1.62 ± 0.86	1.62 ± 0.86	1
Specimen size (cm)	3.82 ± 1.20	3.84 ± 1.37	0.94
Procedure time (min)	98.44 ± 59.89	101.51 ± 68.95	0.688
En bloc resection n, (%)	139 (97.9)	136 (95.8)	0.501
Curative resection n, (%)	138 (97.2)	132 (93.0)	0.169
Continuous antithrombotic agents n, (%)	8 (5.6)	3 (2.1)	0.217

**Table 4:** Hazard ratio for post-ESD bleeding of gastric lavage in propensity score matching cohort.

n = 284	HR	95%C.I.	p
Gastric lavage (+)	0.25	0.071–0.886	0.032

**Fig 2:**
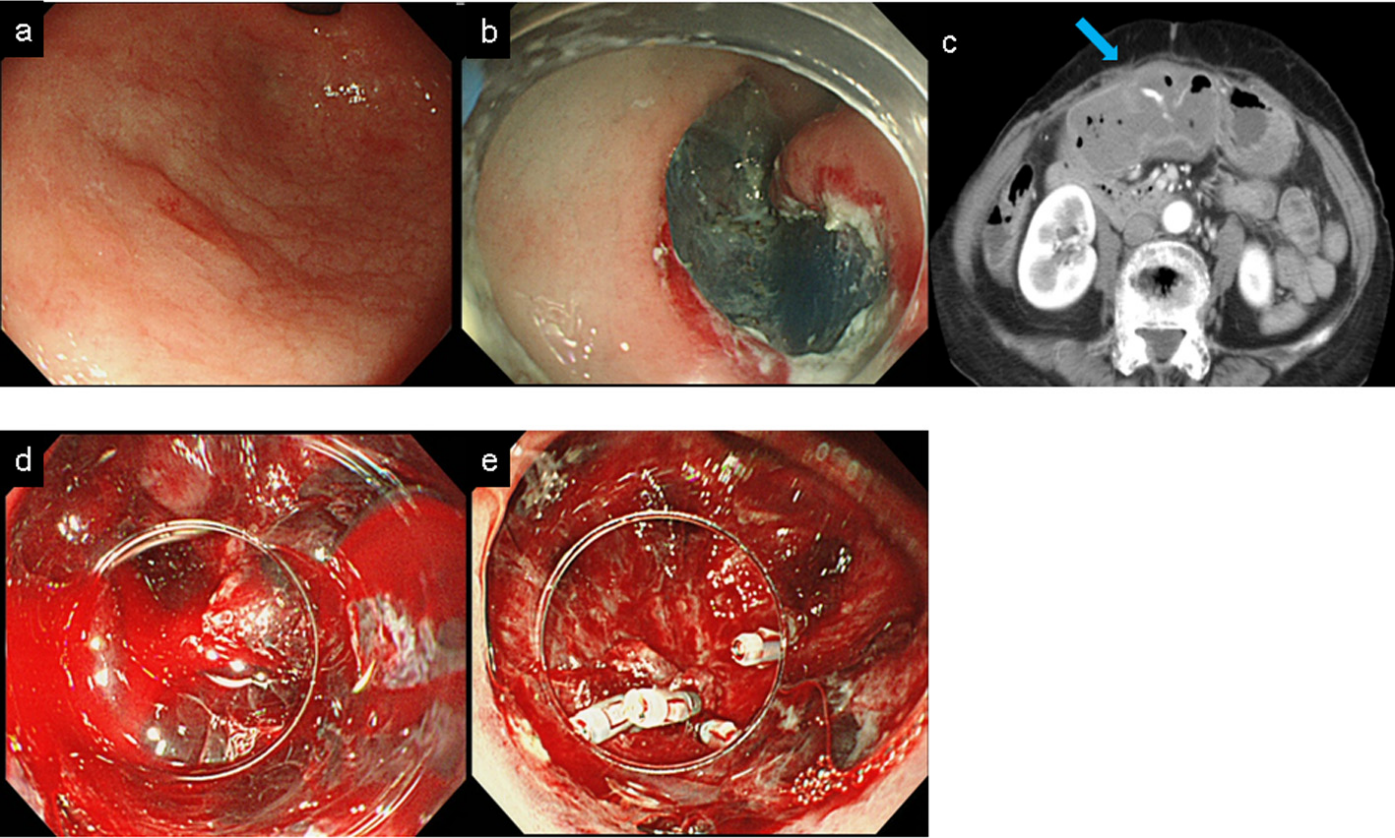
The suggestive case in which postoperative bleeding occurred following the infection in the no gastric lavage group.

A case of postoperative bleeding after ESD in patient without gastric lavage is presented. ESD was performed in 85-year-old woman had a 0.8 cm well differenciated gastric adenocarcinoma in antrum on the anterior wall (Fig 2A and 2B). On 1 postoperative day, the patient had a fever of 38 degrees and hematemesis. Laboratory data showed that the peak white blood cells count was 9700 /μL, the lowest hemoglobin level was 8.7 g/dL, and the peak C-reactive protein level was 4.73 mg/dL. Computed tomography with contrast media showed inflammation of ESD site and spurting bleeding from the post-ESD ulcer, and no evidence of another infectious lesion (Fig 2C). Hemostasis was performed with emergent gastroscopy (Fig 2D and 2E). This case may suggest a possible association between local infection after ESD and postoperative bleeding.

## Discussion

According to recent studies, rates of postoperative bleeding was about 0–15.6%[13]. Postoperative bleeding after endoscopic submucosal resection could be well managed by endoscopic hemostasis. However, emergency endoscopy should be avoided if possible. PPI administration has been recommended to reduce postoperative bleeding after ESD [18]. However gastric bacteria may increase because of an high gastric pH after PPI administration[17], which may become pathogenic infectious agents during ESD. Hence patients who were treated by ESD, have a remarkable risk of infection in the artificial ulcer base because of increased gastric bacteria. And only a larger resection diameter was significant independent risk factor for pyrexia in patients who did not have pneumonia [16]. Mori et al reported that gastric lavage decreases the gastric bacterial count at the end of ESD procedures [17]. They also showed that the increase in white blood cell (WBC) counts on postoperative day (POD) 1 as well as body temperature on POD 2 were suppressed by saline irrigation. This result may indicate that saline irrigation reduced the infection to artificial ulcer as a result of ESD. Therefore it is possible that gastric lavage reduces the infection to ESD site and subsequent complications including postoperative bleeding. We used the tap water for gastric lavage instead of saline. Japanese tap water is drinkable quality according to guideline of the world health organization (WHO) [19]. But it is obscure that preventing infection to ESD site really reduces post ESD bleeding. So the pathophysiology pertaining this issue needs future studies. In the present study we showed for the first time that gastric lavage actually reduced the gastric bleeding after ESD.

This study has some limitations. We analyzed the effect of gastric lavage on post-ESD bleeding by historical control. Except for gastric lavage, the strategy of preventing post-ESD bleeding was not changed. However, the effect of technical improvement or imporved medical appliance could not be completely excluded. Therefore the result needs to be verified in randomized controlled trial.

In conclusion, pretreatment gastric lavage is simple, low cost and readily available. We believe that this knowledgement could improve the safety in ESD procedure.
